# Hypoxia-Driven Extracellular Vesicle Signaling in Lung Cancer Progression, Immune Evasion, and Therapeutic Resistance

**DOI:** 10.3390/antiox15101286

**Published:** 2026-10-03

**Authors:** Dordaneh Mirbabaei Ghafghazi, Panagiota Klentrou, Siu Kwan Sze, Evangelia Litsa Tsiani

**Affiliations:** Faculty of Applied Health Sciences, Brock University, St. Catharines, ON L2S 3A1, Canada; dm19xq@brocku.ca (D.M.G.); nklentrou@brocku.ca (P.K.)

**Keywords:** lung cancer, Hypoxia, extracellular vesicles, EVs, tumor microenvironment

## Abstract

Tumor hypoxia is a defining feature of solid tumors, including lung cancer, and a major driver of aggressive disease progression, metastasis, and therapeutic resistance. Although hypoxia-induced signaling is well-characterized at the cellular level, emerging evidence highlights extracellular vesicles (EVs) as critical mediators through which hypoxic tumor cells remodel the tumor microenvironment. EVs transfer proteins, nucleic acids, and metabolites between cells, enabling complex intercellular communication. Their diverse cargo can act synergistically to promote cancer progression by modulating multiple signaling pathways in recipient cells. In lung cancer, hypoxia enhances EV biogenesis and promotes the loading of oncogenic cargo. Hypoxia-conditioned EVs subsequently drive key cancer hallmarks, including angiogenesis, epithelial–mesenchymal transition, metabolic reprogramming, immune suppression, and therapeutic resistance. Despite the diversity of EV cargo, these effects converge on major oncogenic pathways, including PI3K–Akt, STAT3, Wnt/β-catenin, and PD-1/PD-L1 signaling. Therefore, targeting hypoxia-driven EV signaling may suppress tumor progression and improve treatment efficacy. Clinically, the hypoxia–EV axis offers diagnostic and therapeutic opportunities. Circulating EVs containing hypoxia-associated tumor signatures may support non-invasive liquid biopsy approaches for early detection, prognosis, and treatment monitoring. Targeting EV biogenesis or transfer may also help overcome resistance to targeted therapy and immunotherapy.

## 1. Introduction

Lung cancer is the leading cause of cancer-related mortality globally, accounting for 18.7% of all cancer deaths [1]. Despite major advances in prevention, early detection, targeted therapy, and immunotherapy [1,2], the prognosis for lung cancer patients remains poor, largely due to late-stage diagnosis, aggressive nature, high metastasis potential, and rapid development of therapeutic resistance [3]. These clinical challenges emphasize the need for a more detailed mechanistic understanding of the molecular processes underlying tumor progression and therapeutic failure.

Tumor hypoxia, a common trait of solid tumors like lung cancer, is one of the main causes of these adverse clinical features. Hypoxia arises from rapid tumor growth and abnormal and leaky vasculature, leading to inadequate oxygen delivery [4,5,6,7]. At the molecular level, hypoxia stabilizes hypoxia-inducible factors (HIF-1α and HIF-2α), which translocate from the cytoplasm to the nucleus and bind to hypoxia-responsive elements to regulate transcriptional programs that promote angiogenesis, metabolic reprogramming, immune evasion, and metastatic dissemination [4,5,6,7,8]. Increasing evidence indicates that hypoxia not only drives tumor cell-intrinsic adaptation but also acts as a critical regulator of intercellular communication within the tumor microenvironment [8].

EVs have emerged as key mediators of hypoxia-driven intercellular signaling [9,10,11,12,13]. EVs are lipid bilayer-enclosed, membrane-bound carriers released by cells that range from nano- to micro-scale in size and comprise heterogeneous populations differing in molecular cargo, surface properties, and biogenetic origin, carrying diverse bioactive cargo including proteins, lipids, and nucleic acids that can be transferred to recipient cells to mediate intracellular communication [14,15,16]. These diverse bioactive cargos act synergistically to promote cancer progression by modulating multiple signaling networks in recipient cells. By inducing coordinated molecular reprogramming, EVs provide tumors with an efficient mechanism to drive multiple hallmarks of malignancy [17,18]. Importantly, hypoxia profoundly reprograms EV biology by increasing EV release and selectively enriching their cargo with pro-tumorigenic factors [9,12,19,20,21,22,23,24,25,26]. These hypoxia-conditioned EVs function as systemic signaling units that reprogram cancer, stromal, immune, and endothelial cells both locally and at distant sites, thereby amplifying tumor progression. Since tumor-associated EVs are released into circulation and other bodily fluids (blood, pleural effusions, bronchoalveolar lavage, etc.), they can be isolated and used as a non-invasive and accessible form of liquid biopsy [27] that reflects the molecular state of the tumor and enables early diagnosis and prognostic assessment.

In accordance with current extracellular International Society for Extracellular Vesicles (ISEV) nomenclature recommendations (MISEV2023), the term extracellular vesicles (EV) is used as the default terminology throughout this review. The term “exosome” is reserved for small extracellular vesicles (sEVs) for which an endosomal origin has been experimentally demonstrated. Since the majority of published lung cancer literature characterizes vesicles on the basis of size, molecular markers, or isolation procedures without establishing their biogenesis, such vesicles are referred to here as EVs, even when the original publication describes them as “exosomes”.

In this review, we focus on hypoxia-derived EVs as critical mediators of lung cancer progression. We integrate current and seminal studies to examine how hypoxia modulates EV release and cargo composition and how these EVs contribute to key hallmarks of lung cancer. We first discuss the mechanisms underlying hypoxia-regulated EV biogenesis and cargo selection in cancer cells. Then, we focus on the diverse functions of hypoxia-conditioned EVs in driving multiple lung cancer hallmarks such as angiogenesis, metastasis, cancer stem cell maintenance, metabolic reprogramming, epigenetic modulation, immune suppression, and therapeutic resistance [17,18]. We further explore emerging clinical applications of lung cancer EVs as diagnostic and prognostic biomarkers using liquid biopsy-based approaches. By integrating these recent advances, we aim to clarify the multifunctional roles of hypoxia-conditioned EVs in lung cancer progression and to highlight their potential as targets for early cancer detection and therapy.

This review was updated through 8 September 2026. PubMed was searched using the terms “hypoxia AND lung AND cancer AND (exosomes OR EVs OR sEVs)” to identify primary research articles and relevant reviews. Primary studies were screened by title and abstract for controlled investigations of hypoxia-associated EVs in lung cancer, intermittent-hypoxia or sleep-apnea models, lung-relevant stromal and host-cell studies, therapeutic EV studies, and cross-cancer comparisons. English-language primary studies and major consensus or review articles were included when they informed lung cancer EV biology, hypoxia-conditioned EV signaling, or clinical translation.

## 2. EV Biogenesis and Endosomal Origin of Exosomes

**Figure 1:**
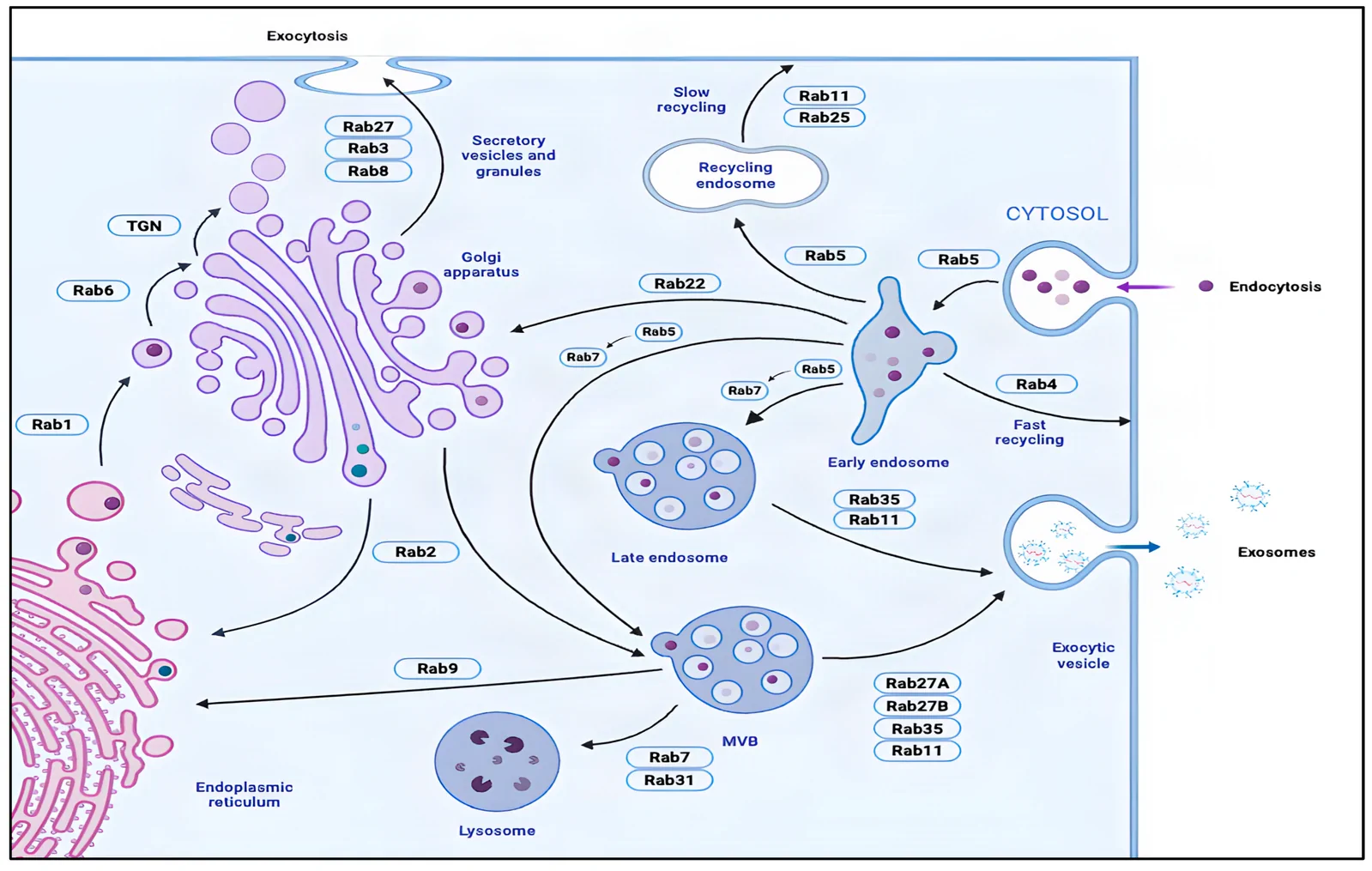
**Rab GTPase-driven pathways of sEV/exosome biogenesis and secretion.** Schematic diagram showing the roles of Rab GTPases in endosomal trafficking routes leading to sEV/exosome formation. Endocytosed membrane and cargo are delivered to early endosomes, a process primarily regulated by Rab5, with Rab4 mediating rapid recycling back to the plasma membrane. Early endosomes mature into late endosomes, and multivesicular bodies (MVBs) form through a Rab5-to-Rab7 transition, during which intraluminal vesicles (ILVs) are formed. Rab35 and Rab11 contribute to ILV biogenesis, cargo sorting, and membrane recycling, linking early and recycling endosomes to MVB maturation and function. MVBs are subsequently directed toward distinct fates: fusion with lysosomes for cargo degradation, regulated mainly by Rab7 and Rab31, or trafficking toward the plasma membrane for exosome secretion. Transport, docking, and fusion of secretory MVBs with the plasma membrane are controlled by Rab27A and Rab27B, with additional contributions from Rab11 and Rab35 in a cell-type-dependent manner. Upon fusion, ILVs are released into the extracellular space as exosomes. In parallel, recycling endosomes, regulated by Rab11, Rab22, and Rab25, mediate slow recycling pathways and intersect with exosome secretion routes. Vesicle trafficking between the endoplasmic reticulum and Golgi/trans-Golgi network (TGN), mediated by Rab1, Rab2, Rab6, Rab8, and Rab9, interfaces with the endosomal system and indirectly modulates the vesicular transport process, thereby influencing downstream exocytic pathways, including those involved at exosome secretion. Created in BioRender. Dordaneh Mirbabaei Ghafghazi. (2026) https://BioRender.com.

EVs are membrane-bound particles released by cells that participated in both physiological and pathological processes, with exosomes representing a distinct population of sEVs [15]. The term “exosome” was first coined in the early 1980s by Trams et al. [28]. Exosome formation is a dynamic and tightly regulated multistep process within the endocytic pathway. Following endocytosis at the plasma membrane, early endosomes mature into late endosomes, which undergo inward budding of the endosomal membrane to generate intraluminal vesicles (ILVs), forming multivesicular bodies (MVBs). MVBs can either fuse with lysosomes for degradation or with the plasma membrane to release ILVs into the extracellular space. This process is primarily regulated by Endosomal Sorting Complex Required for Transport (ESCRT)-dependent mechansims [29,30,31,32,33,34,35]. ESCRT complexes (0 to III) and associated proteins such as Hrs, Tsg101, and Alix mediate the sorting of ubiquitinated proteins and inward budding of endosomal membrane to form vesicles within MVBs [35,36]. Ceramide generation by neutral sphingomyelinase induces endosomal membrane curvature that promotes ILV formation [37], while tetraspanin-enriched microdomains support clustering of specific cargo [38]. Multiple Rab GTPases also regulate MVB trafficking and sEV secretion [33,39,40,41] (Figure 1). Rab7 primarily directs MVBs toward lysosomal trafficking and degradation [39], whereas Rab27a and Rab27b promote MVB docking and fusion with the plasma membrane [41], thereby facilitating sEV release into the extracellular space [41]. Rab27a knockdown has been shown to drastically reduce sEV release, restrict tumor growth, and inhibit metastasis [42,43], implicating sEV-mediated processes in cancer malignancy.

EV function is largely determined by the bioactive cargo they carry. sEV encapsulate a broad and functionally diverse repertoire of molecular cargo, including cytosolic and membrane proteins (enzymes, signaling molecules, receptors, and adhesion proteins), lipids (cholesterol, sphingolipids, and ceramides), and nucleic acids, including mRNAs, microRNAs, long noncoding RNAs, circular RNAs, double-stranded genomic DNA, mitochondrial DNA, and extrachromosomal circular DNA [44,45]. These bioactive molecules retain the molecular state and identity of the cell of origin. The sorting of these molecules into sEVs is a highly regulated process. sEV membranes are enriched in cholesterol and sphingolipids derived from the endosomal membrane, and ceramides in endosomes contribute to negative membrane curvature and inward vesicle budding. The luminal content is composed of cytosolic proteins and nucleic acids [46]. During ILV formation, lung cancer cells selectively package specific molecules into vesicles through mechanisms that include ESCRT-mediated recognition of ubiquitinated proteins (e.g., via Hrs) and interactions with tetraspanin-enriched microdomains. Proteins involved in sEV biogenesis and characterization, such as tetraspanins (CD9, CD63, and CD81), heat shock proteins (Hsc70/Hsp70), Alix, and Tsg101, are therefore commonly detected as sEV-associated proteins. In addition, miRNA loading into sEVs is regulated by defined sequence motifs and RNA-binding proteins such as heterogeneous nuclear ribonucleoproteins (hnRNPs) and Y-box binding protein 1 (YBX1) [47,48]. Tumor-derived sEVs therefore carry complex RNA cargos, including mRNAs, miRNAs, long noncoding RNAs, and circular RNAs, which can reprogram gene expression in recipient cells [12,19,21,22,49,50,51]. They may also contain genomic and mitochondrial DNA fragments, including tumor-specific mutations, effectively encapsulating a molecular signature of the tumor genome [52,53]. Through this selective biogenesis process, EVs can disseminate oncogenic information locally and systemically to distant tissues [7,20,54,55,56,57,58].

**Figure 2:**
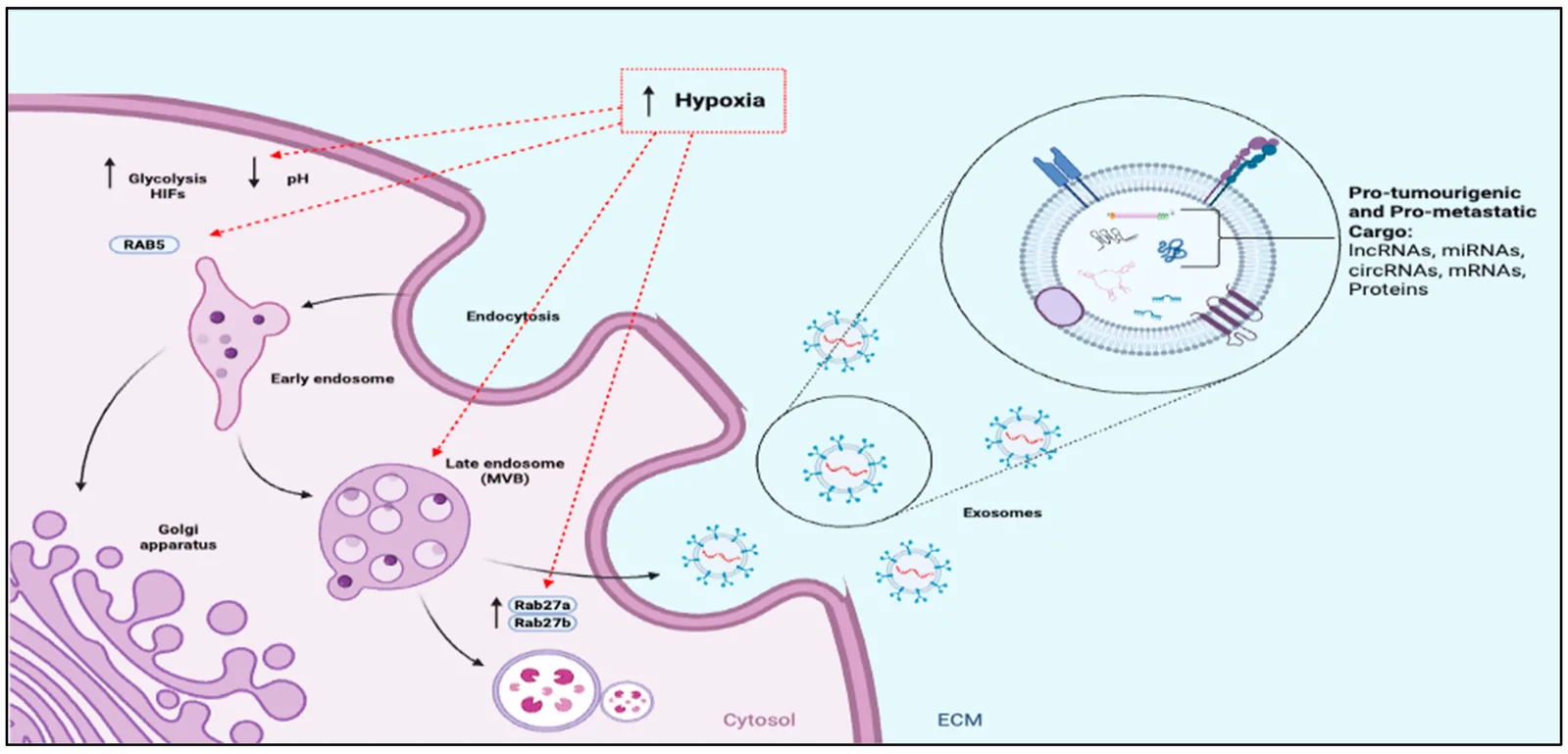
**Hypoxia promotes exosome secretion and selective loading of pro-tumorigenic cargo.** Hypoxic conditions within the tumor microenvironment induce metabolic and signaling adaptations, including increased glycolysis, altered intracellular pH, and activation of hypoxia-inducible factors. These changes modulate endosomal trafficking and vesicle biogenesis, beginning with enhanced endocytosis and early endosome formation, processes associated with Rab5 activity. Early endosomes mature into late endosomes/MVBs, where hypoxia influences ILV formation and cargo sorting. Hypoxia also promotes the trafficking of MVBs toward the plasma membrane and their fusion for exosome release, in part through increased activity of exocytic Rab GTPases, including Rab27A and Rab27B. As a result, exosome secretion into the extracellular space is enhanced. Hypoxia selectively alters exosomal cargo composition, enriching vesicles with pro-tumorigenic and pro-metastatic molecules, including long non-coding RNAs (lncRNAs), microRNAs (miRNAs), circular RNAs (circRNAs), mRNAs, and proteins. Once released, these hypoxia-conditioned exosomes can act on neighboring stromal and immune cells within the TME or disseminate to distant sites, thereby promoting tumor progression, invasion, angiogenesis, immune modulation, and metastatic niche formation. The arrows pointing upwards show increase, while the arrows pointing down show decrease. Created in BioRender. Dordaneh Mirbabaei Ghafghazi. (2026) https://BioRender.com.

Hypoxia in the tumor microenvironment (TME) is a potent upstream regulator of tumour sEV production and cargo loading (Figure 2). A 2010 study demonstrated that hypoxic tumor cells secrete significantly higher levels of sEVs enriched in pro-angiogenic and pro-metastatic cargo [9]. Carcinoma cells cultured under hypoxia released an abundance of sEVs containing bioactive molecules that enhance cell migration and invasion and induce robust angiogenesis in a chorioallantoic membrane assay [9]. Similarly, sEVs derived from hypoxic breast cancer cells were found to be enriched in hypoxia-inducible miRNA miR-210 [10], which promoted angiogenesis, cell migration, and resistance to apoptosis in recipient cells. Such hypoxia-driven changes in sEV-associated cargo have now been documented across multiple cancer types, including alterations in proteins, miRNAs, long noncoding RNAs, and metabolites. The potent oncogenic cargo can synergistically drive EMT, angiogenesis, and immune modulation [12,19,20,21,22]. Mechanistically, hypoxia fundamentally reprograms sEV biogenesis and secretion through HIF-dependent regulation of endosomal trafficking and cargo loading. Stabilized HIF-1α/2α upregulates Rab27a and Rab27b, promoting MVB docking and fusion with the plasma membrane, while concurrently suppressing Rab7-mediated lysosomal trafficking, thereby directing MVB fate toward sEV release rather than degradation [59,60,61,62]. Hypoxia also enhances ILV formation via both ESCRT-dependent sorting and ESCRT-independent pathways involving ceramide generation and tetraspanin reorganization [63]. In parallel, hypoxic signaling selectively enriches sEV-associated cargo, particularly HIF-regulated miRNAs (e.g., miR-210), through RNA-binding protein-mediated loading mechanisms [12,24,64,65,66,67]. Together, these processes increase sEV output and program hypoxia-induced sEVs with pro-metastatic, angiogenic, and immunosuppressive functions. Direct evidence in lung cancer confirms the hypoxia-sEV relationship. Hypoxic lung cancer cells have been shown to markedly increase sEV secretion and to undergo substantial remodeling of sEV-associated cargo composition, with enrichment of pro-metastatic and immunosuppressive molecules [12,26,68,69,70]. sEVs released from hypoxic lung cancer cells carry elevated levels of HIF-regulated factors, such as miR-210 and VEGF, as well as immune checkpoint proteins, including PD-L1 [24,26,66,67,71]. These exosomal cargos collectively stimulate angiogenesis, invasion, metastasis, therapeutic resistance, and immune escape.

**Figure 3:**
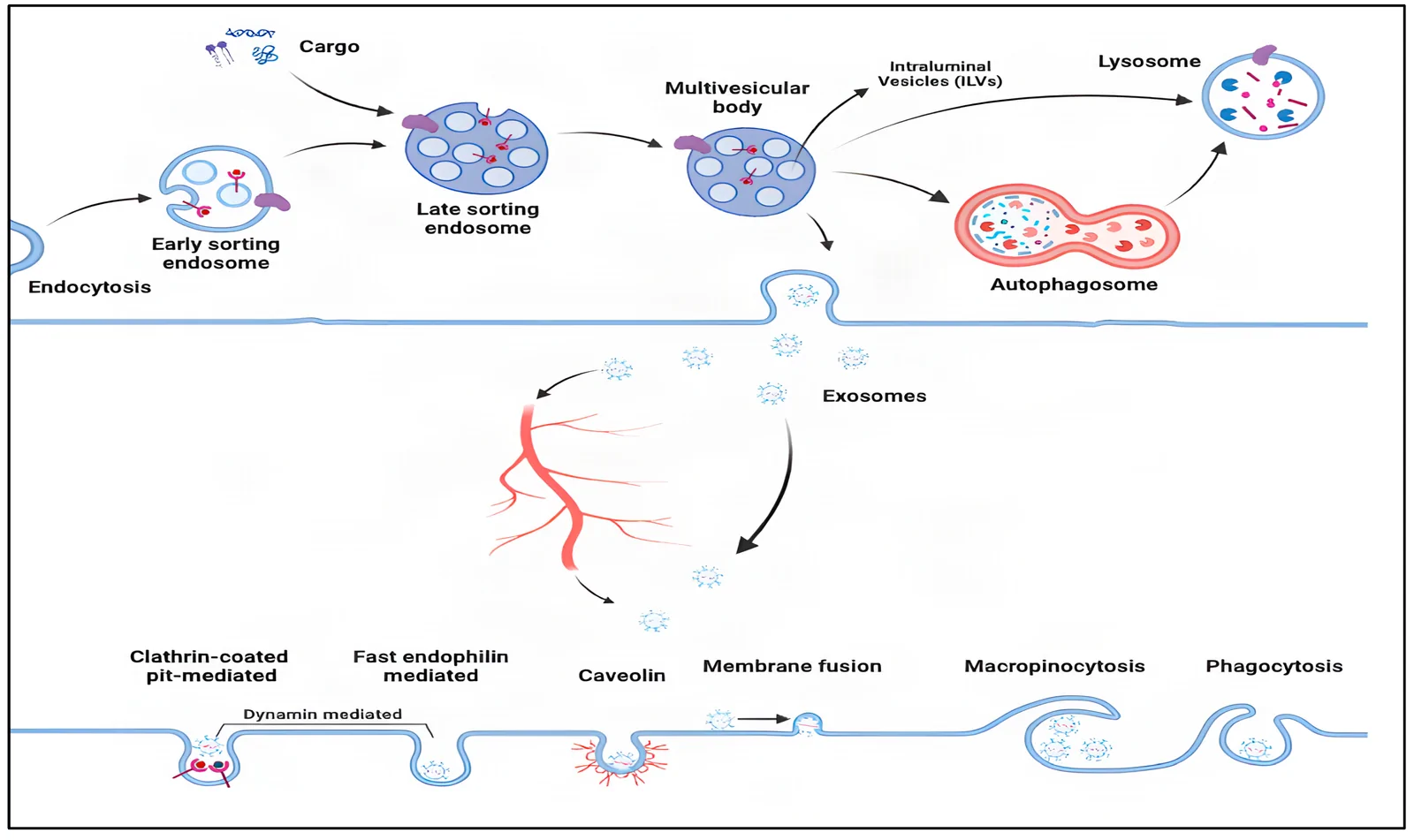
**Exosome biogenesis, secretion, and uptake by local and distant recipient cells.** Exosomes originate from the endosomal system. Plasma membrane components and extracellular cargo from endocytosis are packed into early endosomes, which mature into late sorting endosomes. Inward budding of the endosomal membrane generates ILVs, leading to the formation of MVBs. MVBs have multiple fates: fusion with lysosomes for cargo degradation, interaction with autophagosomes as part of endolysosomal/autophagic pathways, or trafficking toward the plasma membrane. Fusion of MVBs with the plasma membrane results in the extracellular release of ILVs as exosomes. After secretion, exosomes enter the local TME and leak to the circulation to reach distant tissues and organs. Recipient cells internalize exosomes through multiple mechanisms, including clathrin-mediated endocytosis, fast endophilin-mediated endocytosis, caveolin-dependent uptake, direct membrane fusion, macropinocytosis, and phagocytosis. Through these uptake pathways, exosomes deliver bioactive cargo, including proteins, metabolites/lipids, and nucleic acids, to coordinately modulate signaling pathways, cellular behavior, and intercellular communication in both local and systemic contexts. Created in BioRender. Dordaneh Mirbabaei Ghafghazi. (2026) https://BioRender.com.

After release, EVs can travel through interstitial spaces and bodily fluids and be taken up by recipient cells via endocytosis, direct membrane fusion, or receptor-ligand interactions (Figure 3). In lung cancer, tumor-derived EVs are readily detectable in blood, sputum, pleural effusions, and other accessible fluids [72,73,74,75]. Their relative stability in circulation is largely attributable to the lipid bilayer membrane, which protects EV cargo from RNases, proteases, and other degradative enzymes. As a result, EVs are being actively explored as clinically relevant biomarkers. For example, tumor-specific mutations in EGFR and other oncogenes can be detected in plasma-derived EV DNA or RNA, and EV miRNAs and proteins can distinguish lung cancer patients from healthy individuals with high sensitivity [52,53,76]. Beyond their diagnostic and prognostic value, EVs are increasingly recognized as functional mediators of disease progression. They can condition the pre-metastatic niche, promote angiogenesis, modulate immune responses, and transfer drug resistance traits, as discussed in later sections. In summary, EV biogenesis provides lung cancer cells with a powerful mechanism to package and export multifunctional signals that can synergistically reprogram both the local tumor microenvironment and distant tissues to promote disease progression and metastasis. Subsequent sections will discuss how hypoxia regulates EV biogenesis and cargo loading and how the hypoxia-driven EVs promote diverse pro-tumorigenic programs in lung cancer.

## 3. Role of Hypoxia-Associated EVs in Lung Cancer Progression

Hypoxia reprograms EV signaling in multiple hallmarks of cancer by increasing EV biogenesis and enriching pro-tumorigenic proteins, metabolites, and nucleic acids. Hypoxia-conditioned EVs enhance intercellular communication within the microenvironment and at distant sites, facilitating malignant growth, invasion, metastasis, and immune modulation.

In this section, we summarize evidence linking hypoxia-associated EVs to key malignant phenotypes in lung cancer, with emphasis on studies directly using hypoxia-conditioned lung cancer cells. These EVs can alter signaling in cancer, stromal, endothelial, and immune cells through convergent pathways, including PI3K-Akt, STAT3, and Wnt/β-catenin signaling.

### 3.1. Hypoxia-Primed Angiogenic Signaling via EVs

Rapid tumor growth and aberrant vasculature in lung cancer create a hypoxic TME and increase demand for neovascularization. EVs promote angiogenesis by reprogramming endothelial cells within the hypoxic TME [9,25,26,77]. Hypoxic EVs enriched in miR-23a increase endothelial permeability, migration, and angiogenesis by suppressing prolyl hydroxylases 1/2, accumulation of HIF-1α, and inhibition of ZO-1 [77]. EV-associated miR-619-5p similarly promotes endothelial migration, tube formation, and vascular invasion by targeting anti-angiogenic regulators and disrupting endothelial junctions [78]. EVs from hypoxic H1975 lung cancer cells contain elevated levels of miR-671-3p, which influences endothelial angiogenic responses through KLF2 and VEGFR2 regulation, while elevated circulating EV miR-671-3p levels in patients with lung adenocarcinoma further suggest a potential role in pre-metastatic niche formation [79]. These angiogenic mechanisms and representative EV cargos are summarized in Table 1A. Despite diverse cargos, Ev-mediated angiogenesis converges on hypoxia/HIF-1α, PI3K-Akt, Wnt/β-catenin, and TGF-β signaling, coordinating vascular adaptation within the hypoxic TME. Angiogenesis-associated EV cargos may therefore have biomarker potential for hypoxic tumor burden and represent potential therapeutic targets for modulating endothelial reprogramming.

### 3.2. EV-Mediated Proliferation Under Hypoxic Stress

Hypoxia-associated EVs can promote lung cancer cell proliferation and tumour growth through the transfer of regulatory miRNAs and circRNAs. Hypoxic conditions enhance the loading of miR-619-5p into NSCLC-derived EVs, where miR-619-5p suppresses RCAN1.4, promoting tumor-cell proliferation [78]. In addition, hypoxia-associated EV circ_0051799 can promote proliferative reprogramming through circRNA–miRNA interactions that regulate JAK/STAT signaling, contributing to increased proliferation and tumour size [80]. Together, these findings suggest that hypoxia-associated EVs can enhance lung cancer growth through miRNA-mediated suppression of tumor-suppressive regulators and circRNA-dependent signaling reprogramming. The key EV-associated molecules involved in hypoxia-mediated regulation of proliferation and tumour growth are summarized in Table 2A.

### 3.3. Hypoxia-Derived EVs Regulate Cell Death Pathways

Hypoxia-associated EVs can contribute to lung cancer cell survival by suppressing cell-death pathways. Hypoxia-induced EVs have been shown to inhibit ferroptosis through the ANGPTL4/GPX4 axis and to contribute to radioresistance [82]. These findings suggest that hypoxia-associated EV signaling can promote tumor-cell survival by reducing ferroptotic cell death and enhancing resistance to therapeutic stress. The key EV-associated molecules involved in the regulation of apoptosis and ferroptosis are summarized in Table 1C.

### 3.4. Hypoxia-Enhanced EV-Mediated Control of EMT and Metastasis

Hypoxia-driven EVs contribute to metastatic progression by inducing epithelial-mesenchymal transition (EMT), enhancing cell migration and invasion, and conditioning distant pre-metastatic niches [12,84]. Lung cancer EVs carrying miR-582-3p promote invasive behavior by targeting SFRP1 [85]. Hypoxic bone marrow mesenchymal stromal cell-derived EVs can transfer miR-328-3p to lung cancer cells, where it suppresses NF2 and inhibits Hippo signaling, promoting EMT, migration, invasion, and tumor growth [86]. Hypoxic lung adenocarcinoma-derived EVs can also transfer circ-UBR5 to normoxic tumor cells, where circ-UBR5 increases HNRNPR expression and promotes proliferative and invasive behaviors; silencing either circ-UBR5 or HNRNPR attenuates these effects, supporting their role in hypoxia-mediated prometastatic communication [87]. Similarly, hypoxic lung adenocarcinoma-derived EVs enriched in lncRNA FGD5-AS1 promote tumor-cell growth, migration, and invasion through the miR-1179/CDH3 axis [88]. FGD5-AS1 acts as a competing endogenous RNA that limits miR-1179-mediated suppression of CDH3, contributing to the aggressive tumor phenotypes and tumor growth in vivo [88]. Lung cancer-derived EVs can enter the circulation and program distant tissues to establish permissive pre-metastatic conditions [12,89]. Collectively, these findings indicate that hypoxia-associated EVs promote tumor-cell plasticity, invasion, and metastatic colonization through coordinated effects on tumor cells and distant tissues. The key cargos involved in migration, invasion, and EMT are listed in Table 2A.

**Table 2:** Mechanistic summary of EV cargo-mediated regulation of migration, invasion, EMT, cancer stem cells/stemness, and cellular metabolism and oxidative stress in lung cancer under hypoxic conditions. Studies are summarized based on signaling pathways and biological programs, highlighting representative cargos and effects. (**A**) Cell Migration, Invasion, and EMT (Metastasis); (**B**) Cellular Metabolism and Oxidative Stress.

**A. Cell Migration, Invasion, and EMT (Metastasis)**					
**Mechanistic Axis**	**Representative EV Cargo**	**Main Mechanism**	**Key Outcomes**	**EVs Source**	**Reference**
**miRNA-driven EMT, invasion, and metastatic signaling**	miR-328-3p, miR-193-3p, miR-210-3p, miR-5100, miR-619-5p, miR-31-5p, miR-625-3p, miR-106a-5p	miRNA-mediated regulation of STAT3, PTEN, MEK/ERK, WDR5, SCAI, SFRP1, and RCAN1.4	↑ EMT, migration, invasion, N-cadherin, vimentin, β-catenin, in vivo metastatic potential, MMP2, MMP9, Snail ↓ E-cadherin	A549, H1299, BMSC, H460, PC-9, H446, H125, H358, H292, H226	[12,78,85,90,91,92,93]
**circRNA-mediated metastatic signaling**	Circ-UBR5	Circ-UBR5-HNRNPR interaction → enhanced HJNRNPR expression	↑ Migration, invasion, proliferation, and metastatic potential	A549, PC-9	[87]
**lncRNA-mediated metastatic signaling**	FGD5-AS1	FGD5-AS1 → miR-1179 suppression → CDH3 upregulation	↑ Migration, invasion, tumor growth, cell growth	A549, Calu-3, H2110, H23, H69, 16HBE, HSAEC, NuLi-1	[88]
**Epigenetic regulation of EMT**	PKM2	AMPK/p38 signaling → metabolic support for EMT and migration	↑ Migration	A549, H1299, CAFs, H1975	[26,81]
**Cytokine-mediated microenvironmental EMT activation**	TGF-β1, and IL-10	-	↑ Migration	H2228, H1688	[94]
**B. Cellular Metabolism and Oxidative Stress**					
**Mechanistic Axis**	**Representative EV Cargo**	**Main Mechanism**	**Key Outcomes**	**EVs Source**	**Reference**
**lncRNA-mediated glycolytic reprogramming**	OIP5-AS1	OIP5-AS1 → miR-200c-3p regulation → HS3ST1/GPC4-mediated glycolysis	↑ glucose uptake, lactic acid production, ATP levels, glycolysis	A549, H1560	[95]
**Metabolic-redox regulation**	PKM2	Activation of HIF-1α and β-catenin signaling → redox balance modulation and metabolic adaptation	↑ NADH, glutathione ↓ ROS	A549	[83]

Note: The arrows pointing upward demonstrate increase/upregulation while the arrows pointing down show decrease/downregulation.

### 3.5. Hypoxia-Sustained Stemness and Cancer Cell Plasticity

Hypoxia reinforces cancer stem cell phenotypes through EV-mediated transfer of regulatory RNAs and proteins that promote self-renewal, phenotypic plasticity, and therapeutic resilience. Hypoxic bone marrow-derived mesenchymal stem cell EVs containing miR-193a-3p, miR-210-3p, and miR-5100 activate STAT3 signaling and EMT in lung cancer cells [12]. These findings indicate that hypoxia-associated EVs can promote stem-like properties through STAT3- and EMT-related programs, with tumor-stromal EV crosstalk potentially stabilizing CSC states under hypoxic and therapeutic stress. Stemness-associated EV cargos may therefore have potential as biomarkers of minimal residual disease and as therapeutic targets to limit recurrence and improve chemosensitivity.

### 3.6. Metabolic Rewiring Under Hypoxia via EV Signaling

Hypoxia is a key driver of EV-mediated metabolic reprogramming and oxidative stress regulation in lung cancer, reshaping glycolysis and redox homeostasis [81,83]. Hypoxic EVs from cisplatin-resistant NSCLC cells are enriched in PKM2, enhancing its interaction with HIF-1α and β-catenin, reducing ROS levels, and increasing NADH and glutathione levels [83]. These EVs also reprogram CAFs toward a pro-tumorigenic metabolic phenotype [83]. EV-associated lncRNAs, including OIP5-AS1, further enhance glycolytic flux through the HS3ST1-GPC4/miR-200c-3p axis [95]. Thus, EV cargo promotes metabolic plasticity through the regulation of HIF-1α/β-catenin signaling and PKM2-dependent glycolytic control. Metabolically active EV cargos may serve as biomarkers of metabolic vulnerability and treatment resistance, while targeting EV-driven metabolic adaptation could improve therapeutic sensitivity. Table 2B summarizes EV-associated cargos regulating cellular metabolism and oxidative stress.

### 3.7. Epigenetic Reprogramming Driven by Hypoxic EVs

Hypoxia-induced EVs play a critical role in epigenetic reprogramming in lung cancer by transferring regulatory RNAs that reshape chromatin states, RNA methylation, and transcriptional programs [7,12,96,97,98,99]. EVs from intermittently hypoxic bone marrow-derived mesenchymal stem cells transfer miR-31-5p, enhancing EMT, invasion, and tumor growth in vivo via positive regulation of WDR5 [93]. Together, these studies identify EVs as mobile epigenetic regulators in lung cancer. These EV-associated cargos regulating epigenetic reprogramming are summarized in Table 3A.

### 3.8. Immune Evasion Reinforced by Hypoxia-Conditioned EVs

Hypoxia profoundly amplifies EV-mediated immune modulation in lung cancer by reprogramming the tumor immune microenvironment toward immunosuppressive phenotypes [9,25,26,83,100]. Hypoxia-induced EVs promote macrophage recruitment and M2 polarization, with let-7a-mediated metabolic reprogramming enhancing oxidative phosphorylation and suppressing insulin–AKT–mTOR signaling [25]. Hypoxic tumor-derived EVs enriched in PKM2, miR-21, miR-210, miR-1290, and circPLEKHM1 further promote M2 polarization, STAT3 activation, and metastasis [70,81,99,100]. Additional studies identify miR-103a and miR-20a-5p as regulators of hypoxia-associated macrophage reprogramming through PTEN/Akt and STAT30-related signaling, favoring M2-like polarization and immunosuppressive/pro-angiogenic factor production [101,105]. Circulating miR-103a has also been associated with M2 macrophage polarization in patients with lung cancer, suggesting its potential relevance as a biomarker [101]. Similarly, hypoxic NSCLC-derived EVs enriched in SNHG16 promote M2 macrophage polarization through the miR-132-3p/KIF5A regulatory axis and are associated with increased tumor-cell proliferation, EMT, and CSC-related properties [102]. Together, these findings suggest that hypoxia-associated EVs contribute to immune suppression in lung cancer through macrophage recruitment and polarization, metabolic reprogramming, and STAT3-associated signaling, contributing to an immunosuppressive microenvironment that supports disease progression. EV-associated immunomodulatory cargos may therefore have potential as biomarkers of immune dysfunction and resistance to immunotherapy [104]. Targeting EV-mediated reprogramming may enhance antitumor immune immune responses. The bioactive EV molecules involved in immune modulation are summarized in Table 3B.

### 3.9. Therapy Resistance Driven by Hypoxia-EV Crosstalk

Hypoxia is a central driver of EV-mediated therapeutic resistance in lung cancer [7,82,83,106]. Hypoxia enhances EV-mediated transfer of oncogenic RNAs and proteins that promote cancer cell survival, stemness, and immune evasion, and adaptation to therapeutic stress. In NSCLC, hypoxic tumor-derived EVs promote radioresistance by inducing M2 macrophage polarization and increasing TGF-β1 and IL-10 secretion through NDUFV2-associated metabolic reprogramming, characterized by enhanced oxidative phosphorylation and reduced macrophage ferroptosis, which subsequently promotes ferroptosis resistance in neighboring tumor cells [104]. This response is accompanied by increased expression of NDUFV2, GPX4, HIF-1α, Ki67, and CD163 in irradiated tumors, while disruption of NDUFV2 or GPX4 reduces the radioresistant phenotype [104]. Overall, these findings indicate hypoxa-associated EV signaling contributes to therapeutic resistance by coordinating metabolic, immune, and survival responses within the lung TME. The EV-associated cargos involved in resistance to therapy are summarized in Table 3C.

## 4. Other Lung Cancer EV Studies

This section summarizes lung cancer EV studies that did not directly test hypoxia-dependent EV signaling. In many of these studies, oxygen conditions were not reported, or hypoxia was not experimentally manipulated. These studies provide complementary evidence for EV-associated mechanisms in lung cancer but should not be interpreted as direct evidence of hypoxia-driven signaling.

### 4.1. EV-Mediated Angiogenic Signaling

Beyond hypoxia-associated studies, lung cancer EVs have been implicated in angiogenic signaling through diverse RNA and protein cargos. EV-associated miRNAs, including miR-141 and miR-3157-3p, promote endothelial migration and tube formation, while lncRNAs such as GAS5 and MFI2-AS1 regulate angiogenic responses through miR-29-3p/PTEN/PI3K-Akt and miR-107/NFAT5/Akt pathways, respectively [107,108,109,110]. EV-associated circ_0103809, proteins such as LRG1 and PFN2, and M2 macrophage-derived EV miRNAs, including miR-942 and miR-4739, further promote endothelial activation through the miR-153-3p/HDAC1 axis, TGF-β, FOXO1/β-catenin, and Wnt/β-catenin-related pathways [111,112,113,114,115]. These findings provide complementary evidence that EVs can promote angiogenic phenotypes through multiple molecular cargos and signaling pathways. Key examples are summarized in Table 4A.

### 4.2. EV-Mediated Proliferation

Studies conducted without hypoxic conditioning further demonstrate that lung cancer and stromal-cell EVs can promote proliferation through the transfer of regulatory RNAs and proteins. EV-associated lncRNAs, including MALAT1, FOXD3-AS1, and ZBED5-AS1, and circRNAs such as circ-MEMO1, circ_0000519, and circPVT1 regulate proliferation through miRNA-dependent, KRAS, PI3K-Akt, RHO, and EZH2-associated pathways [120,121,122,154,155,156]. TAM- and CAF-derived EVs carrying miR-499a-5p and miR-20a, together with EV-associated proteins such as PFN2 and TRIM59, further support proliferative and clonogenic phenotypes through mTOR, PTEN/PI3K/Akt, and related signaling pathways [113,140,141,168]. These studies support EV-mediated regulation of lung cancer growth but do not establish hypoxia dependence. Representative cargos and pathways are summarized in Table 4B.

### 4.3. EV-Mediated Regulation of Cell Death Pathways

Lung cancer EVs can also modulate multiple cell-death pathways and contribute to tumor-cell survival. EV-associated lncRNAs, including MALAT1, HOTAIRM1, and LINC00662, and circRNAs such as hsa_circ_0014235 and circ_0048856, suppress apoptotic responses through Bcl-2, miR-515-5p-EEF2, miR-613/COMMD8, and miR-320d/E2F1, and related pathways [120,123,130,157,158]. TPX2-containing EVs and stromal EV cargos, including miR-501-3p and miR-20a, further promote apoptosis resistance through Wnt/β-catenin/c-Myc signaling, WDR82, and PTEN/PI3K-Akt signaling [141,142,175]. Reduced EV-associated miR-130b-3p attenuates apoptotic signaling, whereas restoration enhances apoptosis via inhibition of DEPDC1 and TGF-β signaling pathways [173]. In addition to apoptosis, EVs can also regulate ferroptotic cell death. EV-associated miR-4443 suppresses ferroptosis through METTL3/FSP1 signaling in cisplatin-resistant NSCLC [143]. These findings indicate that EV cargo can influence multiple cell-death programs, although they do not directly establish hypoxia-dependent regulation. Representative mechanisms are summarized in Table 4C.

### 4.4. EV-Mediated Control of EMT and Metastasis

Complementary studies without hypoxic conditioning support a broader role for lung cancer EVs in EMT, invasion, and metastatic progression [84]. EV-associated miRNAs miR-106b and miR-1260b have been linked to promoting invasive behavior, with miR-106b targeting PTEN, while reduced miR-34c-3p and miR-130b-3p are associated with enhanced EMT and invasion through integrin α2β1 and TGF-β signaling [49,135,173,176]. EV-associated lncRNAs such as HOTAIR, ZBED5-AS1, and LINC01833, and circRNAs such as circFARSA and circVMP1 further promote EMT and invasion through PTEN/PI3K/Akt, miR-524-5p-METTL3/SOX2, and related pathways [122,124,125,159,177]. Crosstalk with M2-polarized macrophages and systemic EV dissemination may additionally support metastatic niche formation [89,142,178]. These studies provide complementary evidence for EV-mediated metastatic communication. Key EV-associated cargos and pathways are summarized in Table 5A.

### 4.5. EV-Mediated Regulation of Stemness and Cancer Cell Plasticity

Studies conducted without hypoxic conditioning further indicate that EVs can reinforce cancer stem cell-associated phenotypes. Tumor-derived EV circRNAs, including circ_0007385 and circCD226, and EV-associated miR-1246 promote sphere formation, self-renewal, and stemness-associated markers through miR-1253/FAM83A and TRIM17-related mechanisms [160,161,183]. CAF-derived EV-associated METTL3 also enhances stemness in NSCLC cells through m6A modification of SLC7A5 [170]. Collectively, these findings support EV-mediated regulation of self-renewal and stemness through RNA-mediated and epigenetic mechanisms but do not establish a hypoxia-dependent process. Representative cargos are summarized in Table 5B.

### 4.6. EV-Mediated Metabolic Regulation

Complementary evidence indicates that stromal EVs can alter lung cancer metabolism. M2 macrophage-derived EVs carrying lncRNA LINC01001 increase glucose uptake, lactate production, and ATP generation, whereas LINC01001 silencing attenuates glycolytic activity in vivo [186]. This finding provides evidence that stromal EVs can contribute to metabolic plasticity in lung cancer through transfer of regulatory non-coding RNAs. Representative metabolic cargos are summarized in Table 5C.

### 4.7. EV-Mediated Epigenetic Regulation

Lung cancer EVs can also transfer regulatory molecules that influence chromatin and epitranscriptomic states. EV-associated UFC1 and circPVT1 promote malignant phenotypes through EZH2-mediated epigenetic silencing of PTEN, and miR-124-3p/EZH2 further modulates this pathway by regulating EZH2 expression [126,156]. While EV-associated METTL3 promotes m6A modification of SLC7A5 and drives proliferation, invasion, stemness, and glutaminolysis [170]. circVMP1, circRACGAP1, and circ_0103809 further modulate miR-524-5p/METTL3/SOX2 and miR-153-3p/HDAC1 pathways [111,159,184]. These studies support EV-mediated regulation of chromatin modification, RNA methylation, and transcriptional programs but do not directly establish hypoxia dependence. Representative cargos are summarized in Table 6A.

### 4.8. EV-Mediated Immune Modulation

Non-hypoxic studies further demonstrate that lung cancer EVs can modulate immune cells and promote immunosuppressive phenotypes. EV-associated TRIM59 and S100A4 have been linked to macrophage inflammasome activation and STAT3/PD-1/PD-L1 signaling, while EV-associated miR-196b-5p affects T-cell responses and pyroptosis [168,172,194]. M2 macrophage-, CAF-, and NSCLC-derived EV cargoes, including miR-501-3p, miR-181b, miR-1911-5p, miR-146a, miR-155-5p, LINC01001, LINC01833, PCAT6, and PD-L1, have also been associated with macrophage polarization and tumor supportive effects involving proliferation invasion, EMT, angiogenesis, and metabolic reprogramming [18,125,127,142,181,186,191,192,193]. These findings provide complementary evidence for EV-mediated immune modulation without directly establishing hypoxia dependence. Representative cargos are summarized in Table 6B.

### 4.9. EV-Mediated Therapy Resistance

In addition to hypoxia-conditioned EVs, studies without hypoxic conditioning provide complementary evidence that lung cancer EVs contribute to therapeutic resistance. EV-associated H19, hsa_circRNA_102481, circKIF20B, and circ_0076305 have been linked to EGFR-TKI or cisplatin resistance through miR-615-3p/ATG7, miR-30a/ROR1, mitochondrial oxidative phosphorylation, and ABCC1/miR-186-5p [128,164,174,196]. EV-associated miR-210-3p, SNHG7, circDNER, and TPX2 further promote resistance through EMT-related changes, CUL4A-mediated PTEN degradation, miR-139-5p/ITGB8, and Wnt/β-catenin/c-Myc signaling [131,136,165,175]. M2 macrophage-derived EVs can additionally attenuate gefitinib-induced cell death via Akt, ERK1/2, and STAT3 signaling [197]. Collectively, these studies indicate that EV-associated cargos contribute to resistance across targeted therapies and chemotherapy through diverse survival, metabolic, and cellular-plasticity mechanisms, while providing complementary rather than direct evidence of hypoxia-dependent resistance. Representative cargos are summarized in Table 6C.

## 5. EVs as Diagnostic and Prognostic Biomarkers

Lung cancer-derived EVs carry diverse tumor-associated molecular signatures, positioning these vesicles as potential non-invasive biomarkers for cancer detection, prognosis, and therapy monitoring. These EVs contain functional molecules that can influence tumor progression and are released into the TME and circulation. These molecular cargos are protected by a lipid bilayer membrane, which can limit degradation by enzymes in biofluids. Therefore, EVs have substantial potential as non-invasive biomarkers for lung cancer.

Because the molecular cargo of tumor-derived EVs closely mirrors the molecular state of their cells of origin, liquid biopsy approaches based on the analysis of circulating EVs in plasma or serum offer a minimally invasive method to facilitate early cancer detection, disease monitoring, and prediction of treatment response. Key advances in the diagnostic and prognostic applications of lung cancer-derived EVs are summarized below.

### 5.1. EV-Associated Signatures for Early Detection

Circulating EVs enriched with tumor-specific RNAs and proteins, enabling early detection of lung cancer through minimally invasive liquid biopsy approaches. Several studies have identified specific sets of EV-associated RNA in the blood of lung cancer patients. For example, a panel of EV-associated miRNAs (including miR-21, miR-210, and miR-486-5p) has been proposed as a screening tool for early-stage NSCLC, as these are elevated even when tumors are small [12,49,78,84,90,198,199]. Another study showed that miR-1260b levels in serum EVs can distinguish lung adenocarcinoma patients from controls with high sensitivity and specificity [200]. Moreover, DNA mutations (such as EGFR mutations) can be detected in both EV-associated DNA and RNA [136,164,195,199,201]. Encouragingly, assays for the EGFR T790M resistance mutation in plasma often detect a combination of circulating tumor DNA and EV-associated RNA [76], and liquid biopsy-based detection of this EGFR mutant has been established as an FDA-approved companion diagnostic for osimertinib in NSCLC [52,53]. A key advantage of EV-based detection is that EV-associated RNA is of higher quality and more abundant than free-circulating nucleic acids, as they are protected by the vesicle membrane, highlighting the potential of EV-based diagnostics for clinical application.

### 5.2. EV-Associated Markers of Disease Progression

EV-associated molecular cargos can reflect tumor aggressiveness and disease stage, with their molecular cargos serving as prognostic indicators of metastatic progression and patient survival. High levels of several oncogenic EV-associated RNAs have been associated with advanced disease. For example, increased plasma EV-associated miR-21 predicts poorer overall survival in NSCLC and correlates with metastatic burden, consistent with its role in hypoxia-driven invasion and chemoresistance [12,100]. EV-associated MALAT1 is significantly enriched in patients with lymph node metastases and stage III/IV NSCLC and is associated with reduced survival [120,129,130,202]. Similarly, EV-associated circSATB2 correlates with metastatic disease and demonstrates high diagnostic accuracy for lung cancer malignancy [50,147]. Conversely, loss of tumor-suppressive EV-associated RNAs can also indicate aggressive disease; reduced EV-associated GAS5 levels are observed in patients with highly angiogenic tumors [109,132].

In addition to individual markers, several studies have developed multicomponent circulating miRNA signatures for lung cancer detection. For example, a four-miRNA plasma signature (miR-126, miR-145, miR-210, and miR-205-5p) demonstrated high sensitivity and specificity for distinguishing lung cancer patients from cancer-free smokers, independent of tumor stage and histological subtype [51,198].

### 5.3. Monitoring Therapy Response Through EV Biomarkers

EV dynamics provide a real-time window into tumor adaptation during treatment, allowing non-invasive monitoring of therapeutic response and early detection of resistance. Therapy-induced changes in cancer biology are reflected in EV-associated biomarkers that can provide non-invasive monitoring for tumor response to treatment [203]. This approach is particularly valuable for targeted therapies and immunotherapies, where dynamic monitoring of resistance, immune modulation, and tumor adaptation is critical for guiding clinical decision-making. For targeted therapy, a decline in mutant EGFR DNA in EVs after initiating an EGFR inhibitor correlates with tumor response, whereas re-emergence of EGFR mutations or new resistance mutations in MET amplifications or KRAS mutations in EV-associated nucleic acids can signal resistance weeks to months before radiographic progression is evident [204,205]. EV-associated PD-L1 and associated immune-regulatory cargos mirror tumor immune status and may predict response to immune checkpoint blockade therapies. Emerging evidence suggests that EV-associated PD-L1 levels in plasma dynamically reflect tumor PD-L1 expression and total tumor burden [58,197,203,206,207]. Lung cancer patients with high pre-treatment EV-associated PD-L1 had significantly lower response rates to anti-PD-L1 therapy; moreover, EV-associated PD-L1 levels tended to rise in patients who eventually progressed on therapy, possibly indicating an underlying adaptive resistance mechanism [58,197,203,206,207]. Tracking EV-associated PD-L1 could thus help identify patients at risk of relapse during immunotherapy. Additionally, the EV-associated LUCAT1 is a compelling predictive marker in EGFR-mutant NSCLC patients on osimertinib; increasing EV-associated LUCAT1 has been associated with emerging drug resistance [106]. Patients with stable or low EV-associated LUCAT1 maintained longer responses, suggesting it could be used to guide treatment decisions.

## 6. Conclusions and Future Perspectives

Lung cancer is consistently the most lethal cancer type worldwide despite the recent significant advances in cancer prevention, diagnosis, and therapy. This poor clinical outcome is closely linked to its aggressive phenotypes, which are partly driven by its hypoxic TME. Hypoxia profoundly alters tumor EV biology by enhancing EV biogenesis and secretion. Hypoxic lung tumors not only release increased quantities of EVs but are also selectively enriched with pro-angiogenic, pro-metastatic, immunosuppressive, and therapy-resistant cargo. These hypoxia-induced EVs actively drive tumor progression and can be used as accessible biomarkers to assess the tumor’s molecular state. The abundant release of hypoxia-induced EVs presents a unique opportunity for clinical translation. As described above, lipid bilayer-protected tumor-specific molecules in EVs provide a non-invasive approach for early cancer diagnosis, prognostic assessment, and monitoring of treatment response, particularly in targeted therapy and immunotherapy.

Despite this promise, several key challenges remain in harnessing this potential for clinical use. Nearly all cell types in the body secrete EVs, including exosomes, which are highly heterogeneous in size, composition, and cellular origin. In blood and other biofluids, EVs exist as complex mixtures containing diverse populations of vesicles and coexist with other soluble bioactive proteins, lipids, and nucleic acids from multiple sources. Consequently, the isolation and purification of EVs remain technically challenging, underscoring the need for rigorous methodological standardization and careful interpretation of EV-based analyses [14]. Standardization of EV isolation, characterization, and analytical workflows is essential for clinical implementation [14]. Strategies to overcome EV heterogeneity and selectively isolate tumor-derived EVs from complex biofluids are needed for clinical translation. Finally, integrating EV-based assays with existing imaging and molecular diagnostics may provide the most effective approach for early detection, disease prognosis, and monitoring. Continued advances in EV biology and technology are critical to accelerating the translation of EVs from experimental tools to clinically actionable biomarkers and therapeutic interventions in lung cancer.

## Figures and Tables

**Table 1:** Mechanistic summary of EV cargo-mediated regulation of angiogenesis, proliferation and cell-cycle control, and apoptosis and ferroptosis in lung cancer under hypoxic conditions. Studies are summarized based on signaling pathways and biological programs, highlighting representative cargos and effects. (**A**) Angiogenesis; (**B**) Proliferation and Cell-Cycle Control; (**C**). Apoptosis and Ferroptosis.

**A. Angiogenesis**					
**Mechanistic Axis**	**Representative EV Cargo**	**Main Mechanism**	**Key Outcomes**	**EV Source**	**Reference**
**miRNA-driven angiogenesis and vascular permeability**	miR-23a, miR-619-5p, miR-671-3p	HIF-1α signaling; tight junction disruption (ZO-1, occludin); KLF2/VEGFR2 regulation	↑ Angiogenesis, vascular permeability, transendothelial migration, tube formation, blood vessel formation in vivo, VEGF, pre-metastatic niche formation ↓ KLF2	A549, PC-9, H1435, H2030 H460, Calu-1, H1975, HUVECs	[77,78,79]
**B. Proliferation and Cell-Cycle Control**					
**Mechanistic Axis**	**Representative EV Cargo**	**Main Mechanism**	**Key Outcomes**	**EV Source**	**Reference**
**Pro-survival miRNA-Mediated Signaling**	miR-619-5p	miR-619-5p → ↓ RCAN1.4	↑ Proliferation, tumor growth	A549, H460, PC-9, Calu-1	[78]
**circRNA-mediated proliferative reprogramming**	Circ_0051799	circRNA–miRNA sponging → regulation of JAK/STAT	↑ Proliferation, tumor size	A549, H1975, THP-1, H1395, PC-9	[80]
**Epigenetic proliferation**	PKM2	-	↑ Proliferation, tumor weight/volume, Ki67	A549, CAFs	[81]
**C. Apoptosis and Ferroptosis**					
**Mechanistic Axis**	**Representative EV Cargo**	**Main Mechanism**	**Key Outcomes**	**EV Source**	**Reference**
**circRNA-mediated survival and anti-apoptotic signaling**	Circ_0051799	miRNA sponging → JAK/STAT signaling → suppression of apoptotic pathways	↓ Apoptosis, Bax	H1650, serum, PC9, HCC827, SK-MES-1, A549, PC9GR, HCC827GR, H1299	[80]
**Protein-mediated ferroptosis regulation**	ANGPTL4	ANGPTL4/GPX4 axis → oxidative stress regulation	↓ Ferroptosis	A549, H1299	[82]
**Metabolic-epigenetic enzyme axis**	PKM2	PKM2 → glycolytic and AMPK signaling → ROS suppression	↓ Apoptosis, cleaved caspase-3 ↑ Bcl-2	A549, CAFs	[81,83]

Note: The arrows pointing upward demonstrate increase/upregulation while the arrows pointing down show de-crease/downregulation.

**Table 3:** Mechanistic summary of EV cargo-mediated regulation of epigenetic regulation, immune modulation, and therapeutic resistance in lung cancer under hypoxic conditions. Studies are summarized based on signaling pathways and biological programs, highlighting representative cargos and effects. (**A**) Epigenetic Regulation; (**B**) Immune Modulation; (**C**) Therapeutic Resistance.

**A. Epigenetic Regulation**					
**Mechanistic Axis**	**Representative EV Cargo**	**Main Mechanism**	**Key Outcomes**	**EVs Source**	**Reference**
**Epigenetic reprogramming via lncRNA signaling**	mir-31-5p	Regulation of chromatin modifiers (e.g., WDR5) → oncogenic transcription	↑ Malignant phenotypes, EMT, invasion, tumor growth	A549, H1299	[93]
**B. Immune Modulation**					
**Mechanistic Axis**	**Representative EV Cargo**	**Main Mechanism**	**Key Outcomes**	**EVs Source**	**Reference**
**miRNA-mediated immune suppression and inflammatory remodeling**	miR-21 miR-1290 miR-103a	PTEN/AKT/STAT3, SOCS3/STAT3, and IRF1 regulation → M2 macrophage polarization	↑ M2 polarization, NSCLC malignant phenotypes, IL-10, TNF-α, TGF-β	H1299, A549, THP-1, H1437, H1792, H2087, CL1-5	[99,100,101]
**circRNA-mediated immune evasion and macrophage reprogramming**	circPLEKHM1 circ_0051799	EV circRNA signaling → MMP14 stabilization and macrophage M2 polarization	↑ M2 polarization	A549, H1299, PC9, HBE, H1975	[70,80]
**lncRNA-mediated macrophage polarization and immune reprogramming**	SNHG16	SNHG16 → miR-132-3p suppression → KIF5A regulation	↑ M2 polarization, IL-10, TGF-β, VEGF, proliferation, EMT, and CSC-related properties	A549, H1299, THP-1	[102]
**Metabolic control of immune phenotype**	PKM2	AMPK/p38 signaling → metabolic regulation of macrophage polarization	↑ M2 macrophages	A549	[81]
**C. Therapeutic Resistance**					
**Mechanistic Axis**	**Representative EV Cargo**	**Main Mechanism**	**Key Outcomes**	**EVs Source**	**Reference**
**miRNA-mediated chemoresistance**	miR-21	miR-21 → PTEN suppression	↑ Cisplatin resistance, enhanced tumor cell survival under cisplatin treatment	SK-MES-1, A549	[103]
**Metabolic adaptation and therapy resistance**	PKM2, NDUFV2	NDUFV2-associated mitochondrial metabolism → increased OXPHOS → reduced ferroptosis	↑ Cisplatin resistance, M2 macrophage polarization, TGF-β1, IL-10, radioresistance	A549, H1299, THP-1	[83,104]

Note: The arrows pointing upward demonstrate increase/upregulation while the arrows pointing down show decrease/downregulation.

**Table 4:** Mechanistic summary of EV cargo-mediated regulation of angiogenesis, proliferation and cell-cycle control, and apoptosis and ferroptosis in lung cancer. Studies are summarized based on signaling pathways and biological programs, highlighting representative cargos and effects. (**A**) Angiogenesis; (**B**) Proliferation and Cell-Cycle Control; (**C**) Apoptosis and Ferroptosis.

**A. Angiogenesis**				
**Mechanistic Axis**	**Representative EV Cargo**	**Main Mechanism**	**Key Outcomes**	**Reference**
**lncRNA-mediated angiogenesis**	GAS5 MFI2-AS1	miRNA sponging → PTEN/PI3K/Akt, NFAT5/AKT	↑ Tube formation, angiogenesis	[110,116,117,118,119,120,121,122,123,124,125,126,127,128,129,130,131,132]
**miRNA-driven angiogenesis**	miR-23a, miR-619-5p, miR-141, miR-942, miR-4739, miR-375-3p, miR-197-3p	Anti-angiogenic factor regulation; Wnt/β-catenin	↑ Endothelial migration, tube/blood vessel formation	[77,78,107,108,114,115,133,134,135,136,137,138,139]
**Epigenetic regulation**	circ_0103809	miR-153-3p/HDAC1	↑ Angiogenesis	[111]
**Protein-mediated angiogenesis**	LRG1, PFN2	TGF-β signaling	↑ Tube formation	[112,113]
**B. Proliferation and Cell-Cycle Control**				
**Mechanistic Axis**	**Representative EV Cargo**	**Main Mechanism**	**Key Outcomes**	**Reference**
**PI3K/Akt-mediated proliferation**	MALAT1, H19, SNHG7, HOTAIR, miR-20a, miR-3124-5p, miR-141, miR-4739, miR-499a-5p, miR-155-5p	PTEN suppression → PI3K/Akt/mTOR	↑ Proliferation, colony formation, S-phase progression, tumor growth, Ki67	[107,140,141,142,143,144]
**circRNA-mediated proliferation**	hsa_circ_0014235, circRAPGEF5 circPVT1, circVMP1	miRNA sponging → EZH2, CDK4, SOX2, ZEB1	↑ Proliferation, colony formation, S-phase cells, tumor size	[50,145,146,147,148,149,150,151,152,153,154,155,156,157,158,159,160,161,162,163,164,165]
**Oncogenic gene-mediated signaling**	TRIM59, PVRL4, FAM83F	TGF-β1 signaling	↑ Proliferation, tumor growth	[166,167,168]
**Protein-driven proliferative signaling**	PFN2, LRG1	-	↑ Proliferation, tumor growth	[113,169]
**Epigenetic proliferation**	METTL3	SLC7A5/METTL3	↑ Proliferation, tumor growth, Ki67	[170]
**Single-gene mRNA contribution**	ASMA	-	↑ Proliferation	[171]
**C. Apoptosis and Ferroptosis**				
**Mechanistic Axis**	**Representative EV Cargo**	**Main Mechanism**	**Key Outcomes**	**Reference**
**Anti-apoptotic lncRNA signaling**	MALAT1, GAS5, FOXD3-AS1, SNHG7 HOTAIR, LINC00662, HOTAIRM1	PTEN/PI3K/Akt; Bax/Bcl-2; ATR/CHK1	↓ Apoptosis, Bax ↑ Bcl-2	[116,120,121,122,123,124,126,130,131,132]
**miRNA-mediated cell-death regulation**	miR-501-3p, miR-20a, miR-196b-5p, miR-130b-3p, miR-4443	Wnt/β-catenin; METTL3/FSP1; ING5	↓ Apoptosis ↓ Ferroptosis	[141,142,143,172,173]
**circRNA-mediated survival**	circ-MEMO1, hsa_circ_0014235, hsa_circRNA_102481, circVMP1	KRAS, CDK4, ROR1, SOX2 signaling	↓ Apoptosis, Bax	[111,154,157,158,159,164,165,174]
**Gene-mediated tumor survival signaling**	PVRL4	TGF-β signaling	↓ Apoptosis	[166]
**Protein-mediated survival**	TPX2	Wnt/β-catenin/c-Myc	↓ Apoptosis	[175]
**Epitranscriptomic regulation**	METTL3	SLC7A5/METTL3	↓ Apoptosis, cleaved caspase-3 ↑ Bcl-2	[170]
**Single-gene contribution**	ASMA	↓ Apoptosis	-	[171]

Note: The arrows pointing upward demonstrate increase/upregulation while the arrows pointing down show decrease/downregulation.

**Table 5:** Mechanistic summary of exosomal cargo-mediated regulation of migration, invasion, EMT, cancer stem cells/stemness, and cellular metabolism and oxidative stress in lung cancer. Studies are summarized based on signaling pathways and biological programs, highlighting representative cargos and effects. (**A**) Cell Migration, Invasion, and EMT (Metastasis); (**B**) Stemness and Cancer Cell Plasticity; (**C**) Cellular Metabolism and Oxidative Stress.

**A. Cell Migration, Invasion, and EMT (Metastasis)**				
**Mechanistic Axis**	**Representative EV Cargo**	**Main Mechanism**	**Key Outcomes**	**Reference**
**PI3K/Akt and β-catenin-mediated EMT**	MALAT1, H19 HOTAIRM1, LINC00662 HOTAIR, FOXD3-AS1	miRNA sponging → PTEN/PI3K/Akt activation + β-catenin	↑ Migration, invasion, N-cadherin, vimentin, slug, twist, snail, c-Myc, β-catenin, ZEB1, MMP9 ↓ E-cadherin	[110,116,117,118,119,120,121,122,123,124,125,126,127,128,129]
**miRNA-driven EMT/metastasis**	miR-942, miR-4739, miR-210, miR-181b, miR-106b, miR-23a, miR-1260b, miR-3124-5p	TGF-β, Wnt/β-catenin, PI3K/Akt, JAK/STAT, NF-κB, PTEN/KLF suppression	↑ EMT, migration, invasion, N-cadherin, vimentin, β-catenin, in vivo metastatic potential, MMP2, MMP9, Snail ↓ E-cadherin	[134,137,144,179]
**circRNA-mediated EMT**	circ_0014235, circFARSA, circPVT1, circVMP1, circRAPGEF5	miRNA sponging → CDK4, EZH2, SOX2, ZEB1, and JAK/STAT	↑ Migration, invasion, MMP9, N-cadherin, vimentin, MMP2, c-Myc ↓ E-cadherin	[50,145,146,147,148,149,150,151,153,155,156,157,158,159,161,162,165,177,180]
**Oncogenic gene-mediated EMT**	TRIM59, PVRL4, FAM83F	TGF-β1; FAM83F/KIF23	↑ Migration, invasion	[166,167,168]
**Protein-driven migration/invasion**	PFN2, ITGαVβ3, TPX2, PD-L1, LRG1	Cytoskeletal remodeling; ITGαVβ3/FAK; Wnt/β-catenin	↑ Migration, invasion	[113,169,175,181,182]
**Epigenetic regulation of EMT**	METTL3	m6A/SLC7A5	↑ Migration	[170]
**B. Stemness and Cancer Cell Plasticity**				
**Mechanistic Axis**	**Representative EV Cargo**	**Main Mechanism**	**Key Outcomes**	**Reference**
**miRNA-driven stemness**	miR-1246	TRIM17 → stemness programs	↑ Stemness, Oct4, Sox2, Nanog	[183]
**circRNA-mediated stemness**	circ_0007385, circCD226, circSHKBP1, circRACGAP1	miRNA sponging → HMGA2, PKM2, FAM83A	↑ Stemness, Oct4, Sox2, Nanog	[160,161,162,184]
**Epitranscriptomic stemness regulation**	METTL3	M6A/SLC7A5	↑ Stemness	[170]
**C. Cellular Metabolism and Oxidative Stress**				
**Mechanistic Axis**	**Representative EV Cargo**	**Main Mechanism**	**Key Outcomes**	**Reference**
**lncRNA-mediated glycolytic reprogramming**	LINC01001, LUCAT1	METTL3/VEGFA; glycolytic pathways	↑ glucose uptake, lactic acid production, ATP levels, glycolysis	[185,186]
**miRNA-mediated metabolic remodeling**	miR-1290	CUL3 → metabolic adaptation	↑ FAP-1 and FN1	[187]
**circRNA-driven metabolic reprogramming**	circSHKBP1, circCCDC134	miRNA sponging → PKM2, NFAT5	↑ glycolysis	[162,163]

Note: The arrows pointing upward demonstrate increase/upregulation while the arrows pointing down show decrease/downregulation.

**Table 6:** Mechanistic summary of exosomal cargo-mediated regulation of epigenetic regulation, immune modulation, and therapeutic resistance in lung cancer. Studies are summarized based on signaling pathways and biological programs, highlighting representative cargos and effects. (**A**) Epigenetic Regulation; (**B**) Immune Modulation; (**C**) Therapeutic Resistance.

**A. Epigenetic Regulation**				
**Mechanistic Axis**	**Representative EV Cargo**	**Main Mechanism**	**Key Outcomes**	**Reference**
**lncRNA-mediated epigenetic reprogramming**	UFC1	EZH2/PTEN	↑ Malignant phenotypes, EMT, invasion, tumor growth	[126]
**circRNA-mediated oncogenic regulation**	circPVT1, circVMP1, circRACGAP1, circ_0103809	miRNA sponging → EZH2, METTL3/SOX2, HDAC1, RIF1	↑ Malignant phenotypes (growth, metastasis, angiogenesis)	[111,156,159,184]
**Metabolic-epigenetic regulator axis**	METTL3	m6A/SLC7A5	↑ Proliferation, invasion, stemness, glutaminolysis, tumor weight/volume, Ki67	[170]
**B. Immune Modulation**				
**Mechanistic Axis**	**Representative EV Cargo**	**Main Mechanism**	**Key Outcomes**	**Reference**
**lncRNA-driven M2 macrophage polarization**	SNHG7, LINC00963, HOXC-AS2	PTEN/PI3K/Akt, STAT1/SOCS1, ZEB1	↑ M2 polarization, IL-10, IL-23, CCL7, CD206, CD163 ↓ T cell proliferation, MHC-II	[119,125,127,131,186,188]
**miRNA-mediated immune suppression**	miR-196b-5p miR-181b miR-146a miR-155-5p	JAK2/STAT3, PI3K/Akt, ING5	↓ T cells, CD4, CD8, TNF-α, iNOS ↑ IL-1β, IL-18, NLRP3, Treg growth, M2 polarization, NSCLC malignant phenotypes, IL-10, TGF-β	[142,172,178,189,190,191,192,193]
**circRNA-mediated immune evasion**	circFARSA, circSHKBP1, circPVT1	miRNA sponging → PTEN/PI3K/Akt, PKM2	↑ M2 polarization	[149,152,156,162,177]
**Inflammasome activation/cytokine signaling**	TRIM59	NLRP3 inflammasome	↑ IL-1β secretion	[168]
**Protein-mediated immune checkpoint regulation**	S100A4, PD-L1, ITGαVβ3	PD-1/PD-L1, STAT3 FAK signaling	↑ Immunosuppression, M2 macrophages, NSCLC malignant phenotypes ↓ T cell activity	[168,181,182,194]
**C. Therapeutic Resistance**				
**Mechanistic Axis**	**Representative EV Cargo**	**Main Mechanism**	**Key Outcomes**	**Reference**
**lncRNA-mediated drug resistance**	H19, MSTRG.29266, MEG3, SNHG7	PTEN/PI3K/Akt, autophagy (ATG7), CCNE1	↑ Erlotinib resistance, osimertinib resistance, cisplatin resistance, docetaxel resistance	[118,128,131,195]
**miRNA-driven therapy resistance**	miR-210-3p, miR-1260b, miR-96	HIPK2 and hypoxia-associated pathways	↑ Osimertinib resistance, cisplatin resistance, drug resistance	[135,136,137]
**circRNA-mediated multi-drug resistance**	hsa_circ_001423, circVMP1, circ_0076305	miRNA sponging → CDK4, METTL3/SOX2, ABCC1)	↑ Drug resistance, cisplatin resistance, gefitinib resistance, erlotinib resistance, paclitaxel resistance	[157,159,164,165,174,196]
**Protein-mediated resistance**	TPX2	Wnt/β-catenin/c-Myc signaling	↑ Docetaxel resistance	[175]

Note: The arrows pointing upward demonstrate increase/upregulation while the arrows pointing down show decrease/downregulation.

## Data Availability

The data that support the findings of this study are available on request from the corresponding author, S.K.S.

## Abbreviations

The following abbreviations are used in this manuscript. Brief descriptions of their principal biological or physiological functions are provided to aid interpretation.

**Abbreviation**	**Full name and brief functional description**
Akt	Protein kinase B; serine/threonine kinase that regulates cell survival, proliferation, metabolism, and treatment resistance.
ALIX	ALG-2-interacting protein X; ESCRT-associated protein involved in endosomal membrane remodeling and EV biogenesis.
ATP	Adenosine triphosphate; principal cellular energy carrier and phosphate donor.
Bcl-2	B-cell lymphoma 2; anti-apoptotic protein that preserves mitochondrial integrity and promotes cell survival.
CAFs	Cancer-associated fibroblasts; activated stromal cells that regulate extracellular-matrix remodeling, tumor growth, angiogenesis, immunity, and treatment response.
CAIX	Carbonic anhydrase IX; HIF-regulated enzyme involved in cellular pH control and commonly used as a tissue marker of tumor hypoxia.
circRNA	Circular RNA; covalently closed RNA molecule that can regulate gene expression by interacting with miRNAs, proteins, or transcriptional machinery.
CSC	Cancer stem cell; tumor-cell subpopulation with self-renewal, differentiation, tumor-initiating, and treatment-resistant properties.
EGFR	Epidermal growth factor receptor; receptor tyrosine kinase that regulates epithelial-cell proliferation, survival, migration, and differentiation.
EMT	Epithelial-mesenchymal transition; cellular program through which epithelial cells acquire mesenchymal features associated with motility, invasion, plasticity, and treatment resistance.
EV	Extracellular vesicle; lipid-bilayer-enclosed particle released by cells that can transfer proteins, nucleic acids, lipids, and metabolites between cells.
FDA	Food and Drug Administration; United States regulatory agency responsible for evaluating drugs, biological products, medical devices, and diagnostic tests.
GAS5	Growth arrest-specific 5; stress-responsive lncRNA that regulates cell growth, apoptosis, and other context-dependent cellular responses.
H19	H19 imprinted maternally expressed transcript; lncRNA involved in developmental growth, differentiation, and stress-responsive gene regulation.
KRAS	Kirsten rat sarcoma viral oncogene homolog; small GTPase that transmits growth-factor signals controlling proliferation, survival, and metabolism.
lncRNA	Long non-coding RNA; RNA transcript longer than approximately 200 nucleotides that can regulate chromatin organization, transcription, RNA processing, and signaling.
MALAT1	Metastasis-associated lung adenocarcinoma transcript 1; nuclear-enriched lncRNA involved in transcriptional regulation and RNA processing.
METTL3	Methyltransferase-like 3; catalytic component of the principal N6-methyladenosine RNA methyltransferase complex.
miRNA	MicroRNA; short non-coding RNA that generally suppresses gene expression by promoting target-mRNA degradation or inhibiting translation.
MISEV	Minimal Information for Studies of Extracellular Vesicles; consensus guidelines for EV nomenclature, separation, characterization, and functional studies.
MMP2/MMP9	Matrix metalloproteinases 2 and 9; extracellular proteases that degrade basement-membrane and extracellular-matrix components, facilitating tissue remodeling, angiogenesis, and tumor invasion.
mRNA	Messenger RNA; RNA transcript that carries protein-coding information from DNA to the translational machinery.
mTOR	Mechanistic target of rapamycin; serine/threonine kinase that integrates nutrient, energy, growth-factor, and stress signals to regulate growth, metabolism, protein synthesis, and autophagy.
NF-κB	Nuclear factor kappa B; family of transcription factors that regulates inflammation, immune responses, cell survival, and adaptation to cellular stress.
NSCLC	Non-small cell lung cancer; major group of lung cancers that includes adenocarcinoma, squamous-cell carcinoma, and large-cell carcinoma.
PD-1	Programmed cell death protein 1; inhibitory immune-checkpoint receptor that limits T-cell activation following engagement by PD-L1 or PD-L2.
PD-L1	Programmed death-ligand 1; immune-checkpoint ligand that suppresses T-cell activity by binding PD-1 and can facilitate tumor immune escape.
PHD1/2	Prolyl hydroxylase domain proteins 1 and 2; oxygen-dependent enzymes that promote HIF hydroxylation and degradation under oxygen-replete conditions.
PI3K	Phosphoinositide 3-kinase; lipid kinase that activates signaling pathways controlling cell growth, survival, metabolism, migration, and vesicular trafficking.
PKM2	Pyruvate kinase M2; glycolytic enzyme that also performs context-dependent signaling and transcriptional functions.
PTEN	Phosphatase and tensin homolog; tumor-suppressive phosphatase that restrains PI3K-Akt signaling and regulates cell growth, survival, and migration.
ROS	Reactive oxygen species; reactive oxygen-containing molecules that participate in redox signaling but can cause oxidative damage when excessively accumulated.
SCLC	Small cell lung cancer; aggressive neuroendocrine lung cancer characterized by rapid growth, early dissemination, and frequent treatment relapse.
sEV	Small extracellular vesicle; operational term based on physical characteristics, generally including particle size, rather than biogenetic origin.
SOCS3	Suppressor of cytokine signaling 3; negative regulator of cytokine-mediated JAK-STAT signaling.
TAMs	Tumor-associated macrophages; macrophages within the tumor microenvironment that regulate inflammation, immune suppression, angiogenesis, invasion, and treatment response.
TGF-β	Transforming growth factor beta; multifunctional cytokine that regulates growth, differentiation, extracellular-matrix production, immune activity, and EMT.
TME	Tumor microenvironment; cellular and extracellular environment surrounding tumor cells, including immune and stromal cells, blood vessels, extracellular matrix, and soluble factors.
TNF-α	Tumor necrosis factor alpha; inflammatory cytokine that regulates immune activation, cell survival, apoptosis, and tissue inflammation.
TSG101	Tumor susceptibility gene 101; ESCRT-I component involved in endosomal cargo sorting and commonly detected as an EV-associated protein.
VEGF	Vascular endothelial growth factor; secreted growth factor that promotes endothelial-cell survival, vascular permeability, and new blood-vessel formation.
WDR5	WD repeat-containing protein 5; scaffold protein in chromatin-regulatory complexes that contributes to histone modification and transcriptional regulation.
Wnt	Wingless-related signaling pathway; developmental signaling system that regulates cell fate, proliferation, differentiation, stemness, and tissue homeostasis.
